# Use of a Novel Hypercrosslinked Carbohydrate Scaffold for Vocal Fold Medialization in an Ovine Model

**DOI:** 10.1002/oto2.69

**Published:** 2023-10-09

**Authors:** Daniel J. Cates, Yuval N. Nachalon, Amanda L. Johnson, Charles C. Lee, Peter C. Belafsky

**Affiliations:** ^1^ Department of Otolaryngology University of California, Davis Sacramento California USA; ^2^ Comparative Pathology Laboratory University of California, Davis Davis California USA; ^3^ Department of Cell Biology and Human Anatomy University of California, Davis Davis California USA

**Keywords:** dysphonia, glottic insufficiency, regenerative medicine, Scaffold, thyroplasty, tissue engineering, vocal fold medialization, vocal fold paralysis

## Abstract

**Objectives:**

Vocal fold medialization is commonly performed for glottic insufficiency and vocal fold immobility. Currently available materials are temporary injectables or synthetic implants. Acellular scaffolds may allow vocal fold augmentation with autologous tissue via host cell migration. The purpose of this investigation was to evaluate the use of a novel carbohydrate scaffold as a medialization implant.

**Study Design:**

Animal model.

**Setting:**

Academic medical center.

**Methods:**

Unilateral type I medialization thyroplasty was performed in 3 Dorper cross ewes using a hypercrosslinked carbohydrate polymer (HCCP) scaffold. Animals were monitored for 4 weeks for general well‐being, dyspnea, and weight loss. The animals were euthanized at 4 weeks and the larynges harvested. Histologic evaluation was performed to assess for adverse tissue reaction, migration, degradation, and biocompatibility.

**Results:**

No adverse events were reported. No animals lost weight or displayed evidence of dyspnea. Histology demonstrated ingrowth of host cells and neovascularization with minimal peri‐implant inflammatory reaction. Cellular ingrowth into the scaffold was predominately made up of fibroblasts and early inflammatory cells. Scaffold shape was grossly maintained as it underwent degradation and replacement with host tissue. Migration of the implant material was not observed.

**Conclusion:**

Vocal fold medialization in an ovine model with an HCCP scaffold resulted in the ingrowth of host cells with minimal peri‐implant inflammation. Scaffold shape was maintained without evidence of migration as it underwent replacement with host tissue. Further research is required to assess long‐term efficacy in comparison to currently available implants.

Glottic insufficiency is caused by incomplete closure of the vocal folds and can result in hoarseness, difficulty swallowing, and poor cough strength. Glottic insufficiency can result from numerous etiologies including vocal fold paralysis or paresis, vocal fold atrophy, scar, sulcus, and others. The goal of surgical treatment is to improve vocal fold closure by adding bulk to the larynx at the level of the glottis. This can be achieved via injection of viscoelastic filler material or surgical insertion of a synthetic implant into the paraglottic space, displacing the medial edge of the vocal fold. Injection laryngoplasty can be performed in the awake or anesthetized patient, and is ideally suited for the treatment of mild‐to‐moderate glottic insufficiency (glottal gaps < 1‐3 mm).^1^ Early injectables such as paraffin, silicone, and Teflon™ were long‐lasting or permanent, but suffered from poor biocompatibility resulting in inflammatory reactions.^2^ While contemporary injection materials including carboxymethylcellulose, calcium hydroxylapatite, and hyaluronic acid demonstrate substantially improved biointegration,^3^ their inevitable resorption results in clinical effectiveness for only a few months up to 1 to 2 years.^4^, ^5^ Permanent vocal fold augmentation requires open surgical insertion of a nondegradable implant such as Silastic™, Gore‐Tex™, and titanium. The type I thyroplasty approach, first described by Isshiki et al.,^6^ allows a high degree of precision and customization during vocal fold medialization by using simultaneous endoscopic visualization. However, all currently available thyroplasty implants are made of stiff synthetic materials. Despite their placement deep in the paraglottic space, these implants can dampen the vibration of the vocal fold to varying degrees depending on implant stiffness, shape, and placement depth.^7^, ^8^, ^9^ This effect is likely greatest in the setting of vocal fold atrophy, when the thyroplasty implant occupies a greater proportion of vocal fold thickness.^9^ The ideal thyroplasty implant would have biomechanical properties that closely resemble the vocal fold to avoid the vibration‐dampening effect, would be customizable in size, shape, and thickness, and provide a permanent or long‐lasting soft tissue augmentation.

Hypercrosslinked carbohydrate polymer (HCCP) is a novel polysaccharide scaffold developed to facilitate cellular migration and adherence in various organ systems (Figure 1). HCCP has been shown to successfully regenerate critical bone defects and support growth and differentiation of human renal and cardiac progenitor cells.^10^, ^11^, ^12^ The purpose of this investigation was to evaluate the safety and cellular response to a novel HCCP scaffold for vocal fold medialization in an ovine model.

**Figure 1:**
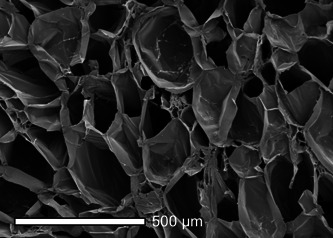
Scanning electron microscope image of hypercrosslinked carbohydrate polymer demonstrating highly porous structure.

## Materials and Methods

### Surgical Procedure

This study protocol was approved by the University of California, Davis Animal Care and Use Committee and institutional guidelines. Three Dorper cross ewes were sedated with a combination of ketamine (10 mg/kg) and midazolam (0.25 mg/kg) injected subcutaneously. Each animal then underwent endotracheal intubation and was mechanically ventilated using inhaled isoflurane (2.0%) to maintain general anesthesia. The ventral neck was clipped and prepped with 10% povidone‐iodine and 70% ethanol alternating scrub, and sterile drapes applied. A 15‐blade scalpel was used to create a 5 cm incision near the inferior border of the thyroid cartilage. The strap muscles were divided in the midline to expose the larynx. A rigid, 0°, 5‐mm telescope was attached to the camera head and video processor (WA50372B, CH‐S190‐XZ‐E/Q, CV‐170; Olympus America Inc.) and was inserted via the oral cavity to visualize the vocal folds. A 25‐guage needle was inserted through the right side of the thyroid cartilage to identify the level of the right true vocal fold, a 4 × 6 mm (*h* × *w*) thyroplasty window was created with a 15‐blade scalpel, and the perichondrium incised to allow access the paraglottic space. Clinical grade 5 mm‐thick sheets of HCCP (Molecular Matrix) were manufactured in a current good manufacturing process facility, sterilized, and tested to meet all product specifications including pore size, porosity, pH, and structural integrity. Each sheet was cut to 6 × 10 × 5 mm (*h* × *w × d*) and inserted through the previously created window into the paraglottic space. Medialization of the vocal fold free edge was visually confirmed using the rigid telescope. Unilateral, right‐side implantations were performed in all animals. The cartilage defect was sealed with bone wax to prevent implant migration. The wound was closed in layers.

Postoperatively the animals were monitored daily per institutional guidelines for signs of general well‐being, pain, and dyspnea. Daily weights were obtained. The animals were allowed to move freely in their pen. The animals were euthanized at 4 weeks with pentobarbital and phenytoin sodium administered intravenously (100 mg/kg). The larynges were excised en bloc and visually inspected. Each larynx was then divided vertically along the anterior commissure for processing.

### Histological Processing and Scoring

Right and left vocal folds of each animal were fixed in 4% paraformaldehyde, embedded in paraffin, sectioned at 2‐μm thickness, and subjected to hematoxylin and eosin (H&E) staining.

Sections were dehydrated using alcohol and cleared using xylenes. Semiquantitative parameters of inflammation were scored by a blinded veterinary pathologist using a commonly used clinical histologic scoring index (Table 1). Ten histologic features were graded on an ordinal scale (0‐4) or rated as present/absent (0/1), for a total possible composite score of 25. Scaffold migration and degradation were assessed at multiple levels.

**Table 1:** Semiquantitative Histologic Inflammation Scoring Index

Histologic feature	Grading scale
Necrosis and degeneration Granulation tissue and fibrosis	0: None
	1: <25% of sample
	2: 26%‐50%
	3: 51%‐75%
	4: 76%‐100%
Granulomatous inflammation Neutrophilic inflammation Lymphoplasmacytic inflammation	0: None
	1: Rare focal or scattered inflammatory cells
	2: 2‐5 small foci per ten 20× fields
	3: 6‐0 small to moderate foci per ten 20× fields
	4: 10+ moderate to large foci per ten 20× fields
Myoregeneration Fibrin Hemorrhage Edema Mineral	0: None
	1: Present

## Results

**Figure 2:**
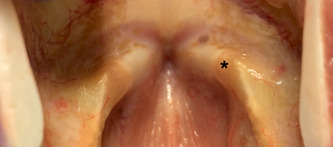
Immediate postmortem inspection of the implanted vocal fold (asterisk) versus control showed no visible signs of inflammation.

**Figure 3:**
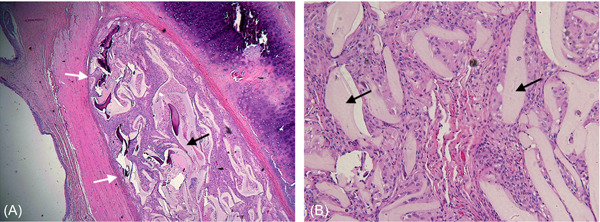
Low‐ (A) and high‐power (B) hematoxylin and eosin‐stained sections through the implanted vocal fold. Residual hypercrosslinked carbohydrate polymer (black arrows) is surrounded by cellular infiltrate, predominately fibroblasts and macrophages, and encapsulated by a thin rim of connective tissue (white arrows).

HCCP was successfully implanted via type I thyroplasty into the paraglottic space. All animals underwent surgery without complication. Once rehydrated with saline or blood, the scaffold became soft but maintained its shape and withstood manipulation without fragmenting. There was no evidence of dyspnea and all animals maintained preoperative weight for the duration of the study. There were no adverse events during routine daily monitoring of animal health. Visual inspection of the larynges at time of euthanasia demonstrated no signs of inflammation, edema, or erythema of either implanted or control vocal folds (Figure 2). Figure 3 displays representative low‐ and high‐power H&E‐stained sections through the implanted vocal fold. At the 4‐week postimplantation time point, histologic analysis demonstrated a consistent reaction in all animals. The implant grossly retained its shape within the paraglottic space despite undergoing near‐complete degradation and replacement with host cells, likely facilitated by the creation of a thin fibrotic capsule. Host cell infiltrate included a predominance of fibroblasts and macrophages with a smaller number of lymphocytes and neutrophils. Although a robust infiltrate of inflammatory cells was evident within the scaffold, very little inflammatory reaction took place in the immediately adjacent tissue layers, indicating a regenerative role of the inflammatory reaction rather than a pathologic response. No migration of implant material was identified outside of the capsule. The blinded semiquantitative total histologic inflammatory score and subscales for implanted and control vocal folds are displayed in Table 2.

**Table 2:** Scoring of Histologic Parameters of Inflammation

Vocal fold	Treatment	Necrosis and degeneration (0‐4)	Granulation tissue, fibrosis (0‐4)	Granulomatous inflammation (0‐4)	Neutrophilic inflammation (0‐4)	Lymphoplasmacytic inflammation (0‐4)	Myoregeneration (0‐1)	Fibrin (0‐1)	Hemorrhage (0‐1)	Edema (0‐1)	Mineral (0‐1)	Total score
1	Scaffold	2	1	4	3	1	1	1	1	1	1	16
2	Control	0	0	0	0	1	0	0	0	0	0	1
3	Scaffold	2	1	4	1	2	0	1	0	1	1	13
4	Control	0	0	0	1	3	0	0	0	0	0	4
5	Scaffold	2	1	4	1	2	1	1	1	1	1	15
6	Control	0	0	0	0	1	0	0	0	0	0	1

## Discussion

HCCP has a well‐established record of safety and efficacy in supporting tissue and organoid growth in preclinical and clinical studies.^10^, ^11^, ^12^ The technology received US Food and Drug Administration approval for bone applications in 2017 with no biocompatibility or safety issues reported after more than 500 patient implantations. HCCP can be manufactured with tunable microporosity and can be cut and shaped at the time of implantation. The scaffold has physical and chemical properties that are unaffected by sterilization using an autoclave. The ideal thyroplasty implant is highly biocompatible, easy to handle, customizable, and closely resembles the biomechanical properties of the vocal folds. The fundamental characteristics of HCCP are in many ways optimized for vocal fold medialization.

The carbohydrate substrate that comprises HCCP is found naturally in the body and undergoes enzymatic degradation to mono‐ and/or oligomeric saccharides. In this study, the majority of the scaffold underwent degradation by 4 weeks postimplantation. While the observed rate of resorption was relatively rapid, the gross implant size and shape underwent little to no change while being replaced by cellular infiltrate. This finding is particularly important in the setting of vocal fold medialization surgery, which requires precision down to a scale of millimeters. Unlike rigid thyroplasty implants, however, due to its inherent degradation it is unlikely that HCCP could be easily explanted after a few weeks in vivo. Rapid degradation of naturally based scaffolds such as HCCP is well‐documented, and it is thought that prolonged residence time of synthetic scaffolds may lead to chronic inflammation and scar tissue formation.^13^ The in vivo structural stability of HCCP appears to be long enough to allow robust host cell ingrowth without losing shape integrity, while minimizing the risk of foreign body reaction. Fibrous tissue deposition throughout the implant comprised of fibroblasts and other connective tissue cells is expected to maintain tissue bulk within the fibrous capsule after full reabsorption of the polymer. The timeline in this study is too short to determine what long‐term tissue augmentation will be generated by the host tissue ingrowth, though early cellular infiltrates are similar to dense fibrous tissue or scar rather than muscle or lamina propria. However, in a study of HCCP implanted into bone defects in rabbit femurs, polymer degradation was nearly complete by 4 weeks postimplantation while new osteoblast formation occurred at the site up to 16 weeks later.^10^ Furthermore, the microporous nature of HCCP allows for tunable rate of scaffold resorption by altering pore size and relative density of the carbohydrate scaffold, which may potentially allow for greater control of degradation time and rate of host cell ingrowth. Cell signaling molecules may easily be added to the porous structure to further manipulate fibrous tissue deposition.

This pilot study was designed to test the safety and feasibility of HCCP implantation into the larynx. All animals were sacrificed at 4 weeks postimplantation as previous studies suggested that most of the polysaccharide construct would be degraded at that time point. This allowed histologic visualization of early tissue ingrowth and screening for inflammatory, pyogenic, or necrotic reactions. However, the lack of histolopathologic data at multiple time points prevents complete understanding of HCCP degradation kinetics and functional tissue development. Further study is needed to investigate whether the volumetric soft tissue expansion effect seen at 4 weeks postimplantation remains stable over time. Additional analysis is required to assess biomechanical effects on vocal fold pliability and vibration. This study provides important pilot data suggesting that HCCP may be a promising alternative to currently available thyroplasty implants. Further investigation evaluating long‐term safety and efficacy is required.

## Conclusion

This study demonstrates that the use of a HCCP for vocal fold medialization is feasible and safe. Implantation is technically straightforward and can be inserted through a standard thyroplasty window. In an ovine model, HCCP scaffold maintains shape stability while undergoing degradation and is mostly replaced by host cells at 1 month. No local inflammatory response or implant migration was observed. Additional preclinical studies are needed to assess the potential for long‐term functional tissue expansion and effects on vocal fold rheometric properties.

## Author Contributions

**Daniel J. Cates**, study design, data acquisition and analysis, manuscript drafting, final approval of manuscript, accountability for all aspects of work; **Yuval N. Nachalon**, study design, data acquisition and analysis, manuscript drafting, final approval of manuscript, accountability for all aspects of work; **Amanda L. Johnson**, study design, data analysis, manuscript review, final approval of manuscript, accountability for all aspects of work; **Charles C. Lee**, study design, data analysis, manuscript review, final approval of manuscript, accountability for all aspects of work; **Peter C. Belafsky**, study design, data collection and analysis, manuscript review, final approval of manuscript, accountability for all aspects of work.

## Disclosures

### Competing interests

C.C.L. is the chief executive officer for Molecular Matrix, Inc., which develops tissue regeneration technology. This article includes similar technology, but no relationship in any form exists with the company.

### Funding Source

The authors have no other funding or financial relationships.
